# Solid Tumors, Liquid Challenges: The Impact of Coagulation Disorders

**DOI:** 10.3390/hematolrep17010008

**Published:** 2025-02-05

**Authors:** Nidha Shapoo, Noella Boma, Shobhana Chaudhari, Vladimir Gotlieb

**Affiliations:** Department of Medicine, New York Medical College, Metropolitan Hospital, New York, NY 10029, USA; boman1@nychhc.org (N.B.); shobhana.chaudhari@nychhc.org (S.C.); gotliebv@nychhc.org (V.G.)

**Keywords:** coagulation disorders, solid, tumors, venous, arterial, thromboembolism, thrombotic microangiopathy, disseminated intravascular coagulation

## Abstract

Coagulation disorders are increasingly recognized as significant complications in patients with solid tumors, affecting morbidity and mortality outcomes. Solid tumors can provoke a hypercoagulable state through the release of pro-coagulant factors, endothelial activation, and inflammation, leading to a heightened risk of coagulation disorders. These coagulation disorders may manifest as venous thromboembolism, arterial thromboembolism, thrombotic microangiopathy, or disseminated intravascular coagulation. These disorders can complicate surgical interventions and impact treatments, including chemotherapy and immunotherapy efficacy, leading to poor outcomes. Understanding the implications of coagulation disorders in solid tumors is essential for optimizing patient management, including identifying high-risk patients, implementing prophylactic measures, elucidating biomarkers for clinical outcomes, and exploring novel therapeutic agents. This review aims to provide insights into the current knowledge surrounding coagulation disorders in solid tumors and their clinical implications.

## 1. Introduction

Coagulation disorders in patients with solid tumors represent a significant clinical challenge, influencing both the course of malignancy and the efficacy of treatment interventions. The interplay between malignancies and the hemostatic system is complex and multifaceted.

## 2. Tumor Biology and Coagulation Activation

**Figure 1:**
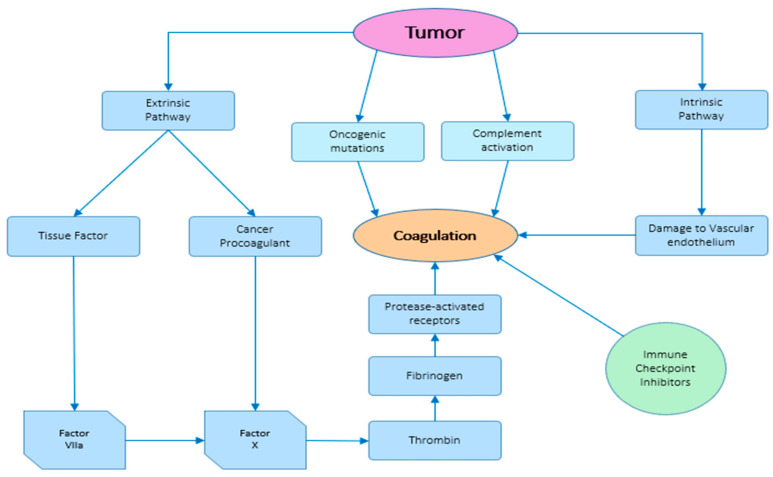
Tumor biology and coagulation activation.

Armand Trousseau first described the association between hypercoagulability and cancer in 1865. The malignant cells express the pivotal clot-promoting factors in activating the coagulation cascade. The key components of the coagulation cascade include the intrinsic pathway, activated by damage to the vascular endothelium, and the extrinsic pathway, mediated by the procoagulant factors [1,2]. The tissue factor is among the most important procoagulant proteins secreted by the cancer cells. Tissue factor (TF), an initiator of the extrinsic coagulation pathway, plays an important role in tumor progression apart from thrombosis. TF is highly expressed in cancer tissues and circulating tumor cells (CTC) and activates factor VIIa, followed by the activation of factor X, leading to thrombin generation. Thrombin exerts its thrombotic effects via the cleavage of fibrinogen and exhibits pleiotropic cellular effects mediated through protease-activated receptors (PARs). Thrombin cleaves and activates PAR-1, -3, and -4 expressed on the surface of many cells, including neurons, fibroblasts, immune cells, platelets, and endothelial cells’ TF and TF-positive microvesicles (TF + MVs), along with stimulating platelets, leukocytes, and endothelial cells which expose their cellular procoagulant features, activating the coagulation cascade. A high TF expression in the tumor tissue of cancer patients has been correlated with thrombosis, tumor progression, and worse prognosis [3]. In addition to tissue factor, some tumor cells can also produce cancer pro-coagulant (CP), which directly stimulates factor X, interfering with the coagulation cascade. Tumor cells also release a variety of soluble pro-inflammatory (i.e., tumor necrosis factor-alpha [TNF-α] and interleukin-1beta [IL-1β]) and proangiogenic (i.e., vascular endothelial growth factor [VEGF] and basic fibroblast growth factor) factors that transform the non-thrombotic and anti-inflammatory endothelial cell surface into an adhesive and procoagulant surface [4]. The complement system’s activation has gained attention recently as a contributor to thrombosis and tumor progression. The activated complement factors C3a and C5a trigger thrombosis by activating platelets and modulating inflammation by impairing CD8^+^ T cell functions. A deficiency of C3 and C5 in mouse models resulted in reduced venous thrombosis. However, the mechanisms of the complement system contributing to coagulation are poorly understood. Immune checkpoint inhibitors have also been associated with thrombotic complications in many clinical studies; however, the underlying mechanisms are not yet known [5]. Some oncogenic mutations have sparked an interest by showing an increased association with thrombosis. KRAS, STK11, ALK, and ROS1 have gained the most attention in solid tumors, and are associated with an increased risk of venous and arterial thromboembolism. The increased risk of thrombosis with these mutations has been seen irrespective of the tumor type. These oncogenic mutations are linked to platelet activation, serine-protease synthesis, tissue factor exposure, and increased thrombin generation, thus leading to hypercoagulability [6,7] (Figure 1).

Various thromboembolic events in solid tumors include venous thromboembolism, arterial thromboembolism, thrombotic microangiopathy, and disseminated intravascular coagulation [8]. In addition to tumor-related factors, patient-related factors, treatment-related factors, and biomarkers increase the risk of thrombosis [9] (Table 1).

Thrombosis can be the first clinical manifestation of a solid tumor and is considered the second leading cause of death in patients with cancer [10,11]. Various studies have shown a significant correlation between the incidence of thromboembolic events and worse prognosis in solid tumors, suggesting the fact that activation of blood coagulation has an impact on tumor metastasis and aggressiveness [12,13,14].

## 3. Solid Tumors and Venous Thromboembolism

Venous thromboembolism (VTE), which encompasses deep vein thrombosis (DVT) and pulmonary embolism (PE), is a significant complication in patients with solid tumors. Patients with cancer have a 4- to 6-fold increased risk of developing vein thrombosis compared to patients without cancer. Approximately 15% of patients with cancer will experience VTE, and conversely, 20% of unprovoked VTEs are the first sign of an underlying malignancy, especially when it develops in less common sites such as the neck or the vena cava, leading to a poor prognosis [15,16]. However, there is little evidence to support routine cancer screening in patients with unprovoked thrombosis. Primary thromboprophylaxis in cancer patients is challenging, as the risk of VTE is not equal in all cancer patients, and anticoagulation is associated with increased bleeding complications in cancer patients. Certain solid tumors like cancer of the pancreas, lung, stomach, uterus, and kidney and brain tumors have an increased risk of VTE [17]. These findings highlight the importance of risk stratification tools, such as the Khorana score introduced in 2007, which aids in identifying ambulatory patients with cancer who may benefit from thromboprophylaxis (Table 2). The Khorana Score was the first risk assessment algorithm to stratify the risk of VTE in cancer patients and has been validated in several retrospective and prospective studies. The risk of VTE in patients with a high-risk Khorana score was 11.0% compared to 6.6% in intermediate-risk and 5.0% in low-risk patients [18].

Other scoring systems have been developed to better stratify patients with cancer according to their VTE risk. The Vienna Cancer and Thrombosis Study (CATS) score is an expanded risk model based on the Khorana score but utilizes D-dimer and soluble P-selectin as additional biomarkers with 1 point assigned to elevated P-selectin (≥53.1 ng/mL) and 1 point to elevated D-Dimer (≥1.44 μg/mL). The risk prediction was considerably improved with CATS score, as the VTE rates were significantly higher in risk groups with a high score than in those with a low score. Patients with ≥5 points had a 35% risk of VTE as compared to 1% in patients with zero points [19].

Munoz A. et al. developed a new score called the ONCOTHROMB score, considering genetic factors and clinical parameters in assessing the risk of VTE in cancer outpatients within 6 months of diagnosis. The ONCOTHROMB score was more sensitive than the Khorana score and better at identifying patients at high risk of VTE likely to benefit from thromboprophylaxis [20].

For primary thromboprophylaxis in ambulatory patients based on risk stratification with the Khorana score, the role of direct factor Xa inhibitors was evaluated in two randomized controlled trials [21,22]. The CASSINI trial evaluated the role of Rivaroxaban, and the AVERT trial evaluated Apixaban in cancer patients with a Khorana score ≥ 2. Based on these trials, most guidelines have recommended the consideration of primary thromboprophylaxis in ambulatory patients with cancer who have a Khorana score of 2 or higher; however, they do not provide knowledge on specific cancer types. In a recent randomized clinical trial (Target-TP trial), a biomarker-driven approach to thromboembolism risk stratification was made, and a significant reduction in thromboembolism and mortality with ambulatory thromboprophylaxis targeted to high-risk individuals with lung or gastrointestinal cancer was found. Target-TP used fibrinogen and D-dimer for risk assessment, and low Molecular Weight Heparin (LMWH) was used as the anticoagulant of choice [23]. The recent American Society of Clinical Oncology (ASCO) guidelines recommend thromboprophylaxis with apixaban, rivaroxaban, or LMWH in high-risk outpatients with cancer (Khorana score ≥ 2 before starting chemotherapy), provided there are no significant risk factors for bleeding and no drug interactions [24].

Anticoagulation is the cornerstone of VTE management in cancer patients. The choice of anticoagulant depends on the patient’s clinical scenario, taking into account cancer type, presence of metastasis, and overall health status. Low Molecular Weight Heparin (LMWH) is the preferred treatment for cancer-associated VTE, especially in hospitalized patients. Direct factor Xa inhibitors such as apixaban or rivaroxaban may be preferred over LMWH for ambulatory patients and those at low risk of bleeding. The duration of anticoagulation therapy for VTE in cancer patients typically depends on the presence of active malignancy and the risk of recurrence. For patients with active cancer and ongoing treatment, the American Society of Hematology guideline panel suggests long-term anticoagulation for secondary prophylaxis (>6 months) rather than short-term treatment alone (6 months) [25].

Apixaban may be an appropriate choice for patients with renal insufficiency due to minor renal clearance [26].

## 4. Solid Tumors and Arterial Thromboembolism

Patients with cancer have a 2-fold increased risk of developing arterial thromboembolism (ATE) compared to patients without cancer. The incidence rate of ATE in cancer patients is estimated to be 2% to 5%, lower than that of VTE. While much of the research has centered on VTE due to its more common occurrence, ATE has gained attention due to its significant morbidity and mortality. Patients with solid tumors are at an increased risk of arterial thromboembolism (ATE). The strongest predictor of ATE risk is the clinical stage and the site of the solid tumor, with metastatic disease and kidney, pancreatic, and lung cancers conferring the highest risks [27]. The risk of ATE is also increased in patients with higher age, male sex, hypertension, and a positive smoking history. Anti-cancer treatments (e.g., radiotherapy, platinum-containing chemotherapy, treatment with monoclonal antibodies, tyrosine kinase inhibitors) are also known to increase the risk of ATE [28]. ATE in cancer is associated with a worse prognosis, a 3-fold increase in overall mortality risk, and a probability of recurrent thromboembolism of 37% at six months [27,28,29].

Arterial thromboembolism typically occurs with endothelial damage. High flow and high shear arterial circulation, in contrast to the low venous shear in the venous circulation, along with the procoagulant materials in the ruptured plaque, lead to the formation of a thrombus [30]. Other factors like marantic endocarditis, secondary antiphospholipid syndrome, tumor embolization, tumor arterial invasion, or tumor arterial compression can also contribute to ATE in solid tumors [31]. Some chemotherapeutic agents like cisplatin and vascular endothelial growth factor (VEGF) inhibitors (bevacizumab, sorafenib, sunitinib, pazopanib) can be prothrombotic [32]. In a recent sizeable genomic tumor profiling registry of patients with solid cancers, alterations in *KRAS* and *STK11* were associated with an increased risk for ATE independent of cancer type [33].

ATE can manifest as acute coronary syndrome (ACS), stroke, or peripheral arterial disease, although the risk of ACS is higher and persists for longer [34]. Sometimes, these may be the first manifestation of solid tumors when their cause is unclear [35]. The risk of cryptogenic strokes is higher (30% vs. 50%) in cancer patients, and a study suggests thrombotic endocarditis as a possible cause of stroke in cancer patients [27]. Another potential mechanism for stroke in cancer is paradoxical embolism, taking into consideration that about 20% of cancer patients develop venous thromboembolism or septic embolism [36].

The management of ATE in patients with solid tumors represents a tough clinical challenge because of the higher risk of bleeding than in the general population, as well as thrombocytopenia and significant drug–drug interactions. The optimal antithrombotic strategy to treat acute arterial thromboembolism in patients with cancer is also uncertain. The diagnosis of ACS in solid tumors follows the same diagnostic algorithm as in general patients. The clinical practice guidelines for managing ACS are derived from observational data and expert consensus. These guidelines propose that invasive approaches for managing ACS are recommended in patients with an expected survival of six months or more. In patients with an expected survival of less than six months or at very high bleeding risk, conservative, non-invasive management strategies should be considered. Since cancer patients are at a higher risk of bleeding, the shortest course of dual antiplatelet therapy is recommended [37]. The management of stroke and peripheral arterial disease follows the same principle as in non-cancer patients.

The primary prevention method for ATE in high-risk cancer patients is not yet known due to a lack of clinical guidelines. In a meta-analysis by Xu Y et al., the use of anticoagulation for the primary prevention of ATE in ambulatory high-risk cancer patients did not show a mortality benefit. Instead, the risk of bleeding was found to be high [38]. Further clinical research is required better to characterize patients with cancer at risk of ATE and explore the role of anticoagulation in the primary prevention of ATE in this high-risk population.

## 5. Solid Tumors and Thrombotic Microangiopathy

Thrombotic microangiopathy (TMA) refers to disorders characterized by microangiopathic hemolytic anemia, thrombocytopenia, and organ dysfunction due to the formation of small blood clots in capillaries and arterioles. While TMA can arise from various causes, its association with solid tumors has gained attention in recent years. The overall incidence of TMA among patients with cancer has been reported to range from 6 to 15%. The exact mechanism by which solid tumors induce TMA is complex and multifactorial. Several factors contributing to this condition include procoagulant substances, such as tissue factor, direct damage to the endothelial cells lining blood vessels, inflammatory cytokines, hypoxia, and platelet activation. TMA can also be triggered by other overlapping conditions such as infections or, more frequently, as an adverse effect of anti-cancer drugs due to direct dose-dependent toxicity or a drug-dependent antibody reaction [39,40].

While TMA can manifest in various forms, the most recognized forms of TMA are thrombotic thrombocytopenic purpura (TTP) and hemolytic uremic syndrome (HUS), which can be acquired or hereditary. A deficit in the activity of ADAMTS13, a von Willebrand factor (VWF) cleaving protease, is the hallmark of TTP. HUS usually results from bacterial infections, particularly *Escherichia coli* O157:H7 (STEC). Atypical HUS (aHUS) is a rare form associated with the overactivation of the complement system, seen in certain solid tumors [41].

Chemotherapy-Induced TMA: Certain chemotherapeutic agents have been associated with the development of TMA, which can be either dose- and time-dependent or non-dose-related (idiosyncratic reactions). Drug-induced endothelial injury is assumed to be the initiating event, but the specific mechanisms remain unclear. Chemotherapy-induced TMA is divided into two categories: Type 1 agents include classical chemotherapeutic agents like mitomycin C, gemcitabine, platinum agents, and proteasome inhibitors. TMA with these agents has a delayed onset, and is irreversible and associated with high mortality. Type 2 agents include vascular endothelial growth factor inhibitors and tyrosine-kinase inhibitors. TMA in these cases can occur at any time and is reversible after interruption of the drug [42].

Tumor-associated TMA is a diagnosis of exclusion, as other conditions like infections, drugs, and chemotherapy must be excluded first. The solid tumors most associated with TMA are gastric, breast, pancreas, prostate, and lung, and the histology mainly involves adenocarcinoma. Usually, patients have advanced cancers when TMA is diagnosed [42].

The clinical spectrum may vary widely from asymptomatic abnormal laboratory tests to acute, severe, potentially life-threatening forms due to massive microvascular occlusion. Patients with TMA often present symptoms indicative of hemolytic anemia, including fatigue, pallor, and jaundice. Thrombocytopenia may lead to easy bruising and bleeding complications. Organ dysfunction is also common, with the kidneys frequently being affected, leading to acute kidney injury. Diagnosis typically involves a combination of laboratory tests, including complete blood count. which reveals hemolytic anemia and thrombocytopenia, and a peripheral blood smear showing schistocytes, elevated lactate dehydrogenase, non-conjugated bilirubin, and reticulocyte count, with reduced haptoglobin suggestive of hemolysis. Coagulation studies are generally normal, distinguishing TMA from disseminated intravascular coagulation (DIC). Patients with solid tumors may present with unspecific symptoms, with classical clinical and laboratory patterns present in only a minority of cases.

Hereditary or primary acquired TMA syndromes like thrombotic thrombocytopenic purpura (TTP), which results from a severe deficiency of ADAMTS13, the most common cause of TMA among adults without cancer, should be excluded. Secondary TMA due to autoimmune diseases, infections, malignant hypertension, and DIC should also be excluded. Measuring ADAMTS13 in cancer may not be helpful, as it could range from undetectable to normal levels, with most patients presenting an adequate level.

The management of TMA in the context of solid tumors requires a multidisciplinary approach. Effective management of the primary malignancy is crucial. Transfusions may be necessary to manage severe anemia or thrombocytopenia. Patients should be monitored closely for signs of bleeding or organ dysfunction. Anti-platelet agents or anticoagulants may be considered, but caution is needed due to the risk of bleeding complications. In patients with tumor-associated TMA, plasmapheresis, steroids, or other immunosuppressive agents used in TTP have no beneficial role. The prognosis of cancer patients with TMA is usually extremely poor due to disseminated cancer [42].

Complement inhibitors, such as eculizumab, have shown promise in treating aHUS and chemotherapy-induced TMA. Eculizumab, a humanized chimeric monoclonal antibody that binds to the human C5 complement protein and prevents the formation of pro-inflammatory C5a and terminal complement complex, led to improved renal and hematological recovery in some patients [39,43].

## 6. Solid Tumors and Disseminated Intravascular Coagulation

Disseminated intravascular coagulation (DIC) is a complex disorder characterized by the widespread activation of the coagulation cascade, leading to blood clots in small vessels throughout the body. This condition can result in multiple organ dysfunction and bleeding due to the consumption of clotting factors and platelets. While DIC can occur in various clinical contexts, its association with solid tumors presents unique challenges in diagnosis and management. DIC occurs in 10–15% of cancer patients and is more commonly a chronic phenomenon with less fulminant presentation than in sepsis. DIC may occur as the first sign of an underlying malignant disease or a late complication of a previously diagnosed and heavily treated cancer [13,44]. Among solid tumors, adenocarcinomas are more prone to trigger both thromboembolic complications and consumption coagulopathy [45]. The hypercoagulability of solid tumors increases the risk of DIC [8]. In solid tumors, DIC is often classified as a secondary form, where the tumor contributes to the dysregulation of hemostasis. The pathogenesis of DIC in cancer includes the derangement of the vascular endothelium in addition to the activation of cytokines and procoagulant factors, such as tissue factor or factor X [46].

Patients may present with signs of thrombosis despite low platelets, including deep vein thrombosis (DVT) or pulmonary embolism (PE), which may not always be readily recognized. Bleeding symptoms may include petechiae, ecchymosis, hematuria, or gastrointestinal bleeding due to the consumption of clotting factors. Thrombocytopenia can lead to multi-organ failure, with renal impairment, hepatic dysfunction, and respiratory distress being common [47].

Diagnosing DIC in the context of solid tumors involves a combination of clinical evaluation and laboratory tests. Key laboratory findings include thrombocytopenia, hypofibrinogenemia, prolonged activated partial thromboplastin time (aPTT) and prothrombin time (PT), and elevated fibrin degradation products (e.g., D-dimer) [48].

A clinical scoring system, such as the International Society of Thrombosis and Hemostasis (ISTH) DIC score, can also aid in the diagnosis. This score includes platelet count and fibrin markers such as D-dimer, PT, and fibrinogen level, with a score over 5 indicating a high likelihood for overt DIC (Table 3) [48].

The management of DIC in patients with solid tumors requires a multidisciplinary approach and is often tailored to the underlying cause, addressing the malignancy through surgery, chemotherapy, or radiation therapy. Successful treatment of the tumor may resolve DIC. Supportive care includes blood product transfusions (platelets, fresh frozen plasma) to manage bleeding and restore hemostatic balance. Anticoagulation may sometimes be indicated, particularly if thrombotic complications are prominent. However, this must be approached cautiously, as the risk of bleeding is significant. Continuous coagulation parameters and clinical status monitoring are crucial in managing patients with DIC, allowing for timely interventions [49].

## 7. Coagulation Factors as Prognostic and Predictive Biomarkers in Solid Tumors

Various coagulation factors have been found to predict outcome in patients with solid tumors, mainly in lung, breast, colorectal, pancreatic, ovarian, and gastric cancers. These include fibrin degradation product (FDP), d-dimer (DD), tissue factor (TF), coagulation factor VIII activity (FVIII), antithrombin III (ATIII), thrombin–antithrombin complex (TAT), α2-plasmin inhibitor–plasmin complex (PIC), thrombomodulin (TM), and tissue plasminogen activator–inhibitor complex (t-PAIC). The elevated peripheral blood levels of these biomarkers were found to have prognostic and predictive value. The coagulation factors may represent promising candidates in future for risk stratification in solid tumors [50].

## 8. Novel Therapeutic Agents and Future Directions

The introduction of direct factor Xa inhibitors has provided more convenient and effective options than LMWH, and they have become the anticoagulant of choice for the treatment and prophylaxis of venous thromboembolism. However, there remains a concern about an increased risk of bleeding in some patients, and also concerns about drug interactions and management in severe renal impairment [51]. Phase II trials have assessed the safety and efficacy of Factor XI (FXI) inhibitors. FXI is a key component of the intrinsic coagulation pathway that plays an important role in cancer-associated hypercoagulability with no disruption in hemostasis, which is primarily an extravascular event. FXI inhibitors that have been investigated include an antisense oligonucleotide, monoclonal antibodies, and a small molecule FXIa inhibitor. All trials compared FXI inhibitors with enoxaparin and found them at least as effective as enoxaparin, with similar safety profiles [52,53,54,55]. Various other trials are ongoing to explore the role of other FX1 inhibitors.

## 9. Conclusions

In conclusion, understanding and managing coagulation disorders in solid tumors is crucial for optimizing patient care. Clinicians must adopt a proactive approach to assess thrombotic risks, implement appropriate prophylactic measures, and consider the implications of anticoagulant therapy in the context of cancer treatment. By addressing these challenges, healthcare providers can enhance the quality of care for patients with solid tumors and mitigate the associated risks of coagulation disorders. Future research should focus on elucidating the underlying mechanisms of coagulation disorders in different tumor types, addressing biomarkers for risk stratification, and exploring novel therapeutic strategies that consider safety profiles.

## Figures and Tables

**Table 1:** Factors associated with thromboembolism in solid tumors.

**Tumor-related factors** ○ Tumor site: highest risk with pancreas, stomach, lung, kidney, uterus, and brain tumors. ○ Stage of cancer: highest risk with metastatic disease. ○ Time from diagnosis: highest risk in the first 3 months of diagnosis. ○ Genetic mutations: KRAS, STK11, ALK, ROS1.
**Patient-related factors** ○ Obesity. ○ Ethnicity: More prevalent in Black Americans. ○ Prolonged immobility. ○ Family or previous history of thromboembolism. ○ Inherited thrombophilia disorders. ○ Comorbidities: renal disease, respiratory disease, acute infections.
**Treatment-related factors** ○ Surgery. ○ Chemotherapy: platinum-based drugs like cisplatin. ○ Immunomodulatory drugs: thalidomide, lenalidomide. ○ Vascular endothelial growth factor inhibitors like bevacizumab, sunitinib, sorafenib, and pazopanib. ○ Immune checkpoint inhibitors. ○ Erythropoiesis-stimulating agents. ○ Blood transfusions. ○ Central venous catheters.
**Biomarkers** ○ High D-dimer levels. ○ Pre-chemotherapy low hemoglobin, high leukocyte count, and high platelet count.

**Table 2:** Khorana risk score.

Variable	Score
Very high-risk tumor (stomach, pancreas)	2
High-risk tumor (lung, gynecologic, genitourinary (excluding prostate))	1
Hemoglobin level < 100g/L or use of red cell growth factors	1
Prechemotherapy leukocyte count > 11 × 10)9/L	1
Prechemotherapy platelet count 350 × 10)9/L or greater	1
Body mass index 35 kg/m^2^ or greater	1

A score of 0—low-risk category, 1–2—intermediate-risk category, and >2—very high-risk category.

**Table 3:** International Society of Thrombosis and Hemostasis (ISTH) DIC score.

Test	0 Points	1 Point	2 Points	3 Points
**INR or** **PT prolongation**	INR ≤ 1.3 <3 s	INR 1.3–1.7 3–6 s	INR > 1.7 >6 s	
**Fibrinogen**	>100 mg/dL	<100 mg/dL		
**D-dimer**	<400 ng/dL		400–4000 ng/mL	>4000 ng/mL
**Platelets**	>100,000/uL	50,000–100,000/uL	<50,000/uL	

≥5 points: Overt DIC. INR-International normalized ratio, PT-prothrombin time.
